# Providing Aging Adults Social Robots' Companionship in Home-Based Elder Care

**DOI:** 10.1155/2019/2726837

**Published:** 2019-06-04

**Authors:** Na Chen, Jing Song, Bin Li

**Affiliations:** ^1^School of Economics and Management, Beijing University of Chemical Technology, Beijing, China; ^2^School of Economics and Management, Tsinghua University, Beijing, China

## Abstract

Population aging is increasingly serious. The application of social robots for home-based elder care is an important way to solve this problem. Aging adults' demands for social robots' companionship affect robotic designs. This study aimed to investigate aging adults' demands for social robots' companionship and explored in which life situations it was appropriate to accompany aging adults by social robots. This study involved three phases. Phase 1 (an interview survey) collected the life situations in which aging adults lived alone at home. Based on the results of Phase 1, Phase 2 (a questionnaire survey) investigated aging adults' demands for companionship, whereas Phase 3 (an expert evaluation) investigated the feasibility of the robots' companionship for aging adults. After the three phases, this study compared aging adults' demands for companionship with the feasibility of social robots' companionship in each life situation. Based on the results, the life situations of dinning and watching TV, there was a greater likelihood that the companionship that aging adults needed might be provided by social robots. In the life situations of sleeping and short breaking, it was difficult that aging adults' demands for companionship were fulfilled by social robots. Implications were discussed for home-based elder care system.

## 1. Introduction

The aging population of the world is rising rapidly. In 2017, the number of the population over the age of 60 years old has reached 962 million, making up 12.7% of the world population; by 2050, this number would double to approximately 2.1 billion, making up 21.4% of the world population [1]. Population aging is a complex issue that concerns not only the well-being of the aging adults but also brings social and economic problems. The adoption of robots to provide elderly services is a crucial way to solve the series of social and economic problems brought about by population aging. How robots can support aging adults' life has already attracted the interests of a large number of researchers [2]. The possibilities of robots in home-based elder care improve the quality of aging adults' life, especially the younger aging adults, alleviate the pressure on institutional elder care, and is in line with the tradition of some countries that prefer home-based elder care, such as China.

With the development of science and technology, robots are not only considered as assistive devices providing physical services, they are also perceived as social agents providing a growing variety of social services, such as entertainment [3, 4], communication [5], and comfort [6]. The robots with social functions are defined as social robots [7]. The companion function is an important development direction of social robots [8], which provide aging adults psychological comfort so as to reduce their feelings of loneliness, depression, and social isolation [9, 10]. However, the research exploring social robots' companionship, especially aging adults' subjective demands for companionship, is still relatively inadequate. This study explored aging adults' demands for companionship of social robots based on specific service scenarios.

Among the research on providing aging adults companionship in home-based elder care, some research teams develop and investigate companion robots to assist aging adults, such as Paro [11, 12] and Huggable [13, 14]. Paro and Huggable only have the function of a social companion without any assistive functions and have the same beneficial effects on aging adults that real animal pets could have, such as making aging adults happier and healthier. The development of these companion robots follows the general sense that pets can accompany aging adults. Some research suggests that pet ownership may reduce loneliness of aging adults who live alone [15]. However, keeping animals causes some issues, such as animal ethics and feeding issues. Social robots with the companion function can largely avoid the disadvantages generated by keeping animal pets at home [16]. They can serve as pets and fulfill roles that caregivers would fulfill in rehabilitation [8].

There is still no clear definition of social robots' companionship. Some research involves companion behaviors [17–20]. However, the companion behaviors of social robots in the research are relatively equivalent to social behaviors, such as showing competence, showing responsibility, and expressing empathy. This study first distinguished between social behaviors and companion behaviors. Based on Ohya's [21] study, this study defined social robots' companionship as maintaining a range of close spatial distance with the users (aging adults in this study) during the service and enabling the users to perceive their presence and companionship.

Although social robots have advantages in addressing home-based elder care, there are still many issues with the interaction between social robots and aging adults. On the one side, the cognition of the majority of aging adults about intelligent robots is deeply influenced by science fiction movies and novels; this leads to that they have illusions about the functions of robots and not to know the actual levels of robotic technology [22]. In addition, it is hard for aging adults who have no experience with social robots to imagine their interaction scenarios. On the other side, aging adults are not always willing to accept new technologies and it might be crucial to determine their subjective demands for social robots' companionship [8]. Hence, research should be conducted to investigate aging adults' demands of social robots' companionship and the feasibility of the companionship in order to provide design recommendations of social robots' interactive behaviors, to establish a well-developed human-robot interaction system, and to provide better home-based elder care services for aging adults.

Based on the facts that aging adults have no adequate understanding of social robots and it is hard for them to imagine the interaction with social robots, studies on the interaction between aging adults and social robots should be focused on specific life situations in which social robots accompany aging adults so as that the aging adults feel immersed in the situations and fully realize the companionship of social robots. However, few research studies focus on social robots' companionship based on specific life situations. The life situations this study focused on involved the ones in which aging adults can live alone, such as dining and studying.

This study aimed to investigate aging adults' demands for companionship in specific life situations in which they live alone as well as the feasibility of social robots' companionship in these life situations and compared the demands and feasibility to explore the interchangeability of social robots and human beings while they provide aging adults' companionship. This study involved three phases. Phase 1 was an interview survey—through an in-depth interview with aging adults—to identify their current life status, to understand their life situations they often experience when they live alone, and to learn their understanding and perception of the companionship. Based on the collection of the life situations in Phase 1, a questionnaire survey (Phase 2) and an expert evaluation (Phases 3) were conducted. The questionnaire survey investigated the life situations in which aging adults need companionship. Because of the limitation of aging adults' imagination for social robots' companionship, an expert evaluation was conducted to assess the feasibility of social robots' companionship for aging adults. The results of Phase 2 and Phase 3 were compared to explore the possibility of replacing human beings' companionship with social robots'.

## 2. Phase 1: Interview Survey

### 2.1. Participants

This study recruited eight aging adults as interviewees from an elderly university in Beijing. Their average age was 66.8 (SD = 3.99), ranging from 60 to 72 years old. Four of them were female and the others were male. Three of them held high school degrees, five of them held undergraduate degrees, and the others held master degrees or above. They all had ever seen or even used robots. Two questions using a 5-point Likert scale was asked to test their computer skills and understanding of robots, respectively. Three interviewees thought they had slightly advanced computer skills, four interviewees thought they had neutral computer skills, and the others thought they had slightly poor computer skills. One interviewee thought he/she had a slightly sufficient understanding of robots, six thought they had a neutral understanding, and the others thought they had a slightly insufficient understanding.

### 2.2. Interview Questions

In order to investigate aging adults' life status and their demands for companionship, the interview questions mainly included the following aspects: (1) the interviewees' current life situations and their experience of living alone at home; (2) their experience of inconvenience or loneliness that they felt in family life; (3) whether they need to be accompanied; (4) what are their demands for companionship; (5) their experience of interaction with robots.

During the interview, interviewers should take care of aging adults' self-esteem and respect their mentality that they did not admit they were aging.

### 2.3. Results

Based on the results of interview, twelve life situations in which aging adults live alone at home were summarized, including dinning, doing housework, doing healthcare by selves, exercising, watching TV, sleeping, using computers/telephones, reading, doing religious activities, entertaining, studying professional skills/knowledge, and short breaking. Among the life situations, some interviewees reported that they had pedicure, massage, and scraping (a popular treatment for sunstroke by scraping the patient's neck, chest, legs, and other body parts) by themselves for healthcare or treatment; these activities were recorded as doing healthcare. Some interviewees did religious activities, including coping Buddhist scriptures or praying to God. Some interviewees studied professional skills, such as calligraphy and English. The frequencies with which these situations occur and the frequencies with which aging interviewees demand companionship are listed in detail in Table 1. The frequencies with which these situations occur and the frequencies with which aging interviewees demand companionship are basically positively correlated, except for the life situation of sleeping in which interviewees reported high occurrence frequency but low demand frequency.

According to the results of the interview, the life situations in which aging adults' demands for social robots' companionship had three features as follows:

1. Life situations in which they do not need to concentrate or pay much efforts to accomplish tasks, such as doing housework and doing healthcare; in these life situations, aging adults had stronger demands to be accompanied
2. Life situations in which they had desire to share they ideas; at this time, aging adults wanted to be accompanied by social robots because the robots can listen to their talk
3. Life situations in which they felt lonely, such as the moments they just waked up

## 3. Phase 2: Questionnaire Survey

### 3.1. Participants

According to the retirement age regulated by the Chinese government, the general retirement age of Chinese women is 55 years old and that of Chinese men is 60 years old; this study took the aging adults aged from 57 years old to 74 years old as younger aging adults [23] and took them as research subjects (potential participants).

Participants in this study were recruited from the senior centers of two communities and one university for aging adults in Beijing. A total of 431 questionnaires were collected. Among them, 234 questionnaires were valid—all items in the questionnaires were filled, the answered were not obviously the same, and the ages of the responders were in the range of 57–74 years old. Among the valid responses, the average age was 63.67 years old (SD = 4.72). Sixty-three responders were male, making up 26.92% of the total, whereas the other 172 were female. Nine responders held master or above degrees, 51 responders held undergraduate degrees, 97 responders held high school degrees, and the others held low-level degrees.

### 3.2. Instrument

This phase was conducted to investigate in which life situations collected in Phase 1 aging adults needed companionship. The questionnaire items were developed based on the Comfort from Companion Animals Scale [24]. The original scale consists of 11 items. Considering that the aging adults should answer the items according to each of the twelve life situations—this leaded to a heavy work and some items in the original scale were not suitable for this study—three items were selected and revised according to the research aims of this study. The remaining items involved “I need companionship in this life situation when I live alone,” “I need someone to love in this situation when I live alone,” and “I need something to care for in this situation when I live alone.” Each item was measured with a 5-point Likert scale with “1 = strongly disagree” and “5 = strongly agree.”

### 3.3. Results

The descriptive statistics for the aging adults' demands for companionship in each life situation are presented in Table 2. In order to verify interreliability between items for the scales, Cronbach's alpha values were determined for these constructs. In general, alpha values of at least 0.6 are considered acceptable for short instruments with a small number of items [25]. The constructs of all the life situations had acceptable alpha values as shown in Table 2.

Aging adults' demands for all life situations were ranked according to the average score of the constructs and are shown in descending order in Table 2—the higher the score, the stronger the demand of the aging adults for companionship in this life situation. The top one-third life situations in the ranking list were considered as high-demand ones, involving dining, doing housework, exercising, and doing healthcare; this suggested that aging adults had stronger demands for companionship in these life situations. The middle one-third life situations were considered as medium-demand ones, involving using computers/telephones, studying professional skills, watching TV, and entertaining; this suggested that aging adults had medium demands for companionship. The last one-third life situations were considered as low-demand ones, involving doing religious activities, short breaking, reading, and sleeping; this suggested that aging adults had weak demands for companionship.

## 4. Phase 3: Expert Evaluation

### 4.1. Methods

The life situations in which aging adults live alone at home are potential service scenarios in which social robots provide companionship. Based on the life situations collected in Phase 1, this study conducted an expert evaluation to assess the levels of the feasibility of the companionship provided by social robots.

Five experts in the field of elderly study were invited, including three associated/assistant professors and two senior researchers. Among them, two experts' research fields cover mainly human-computer interaction, one expert's research field covers mainly human-robot interaction, and the other two experts' research fields cover mainly elderly study. The three experts whose research fields cover human-computer/robot interaction also ever did research related to aging adults. Hence, the five experts had sufficient knowledge highly related to this study and could fully understand the purpose, research methods, and contents of this study. They could provide an evaluation with high levels of reliability and credibility.

The evaluation method referred the Delphi method [26] and took the back-to-back method—the five experts did not have any contact with each other and just communicated with experimenters through e-mail. The experimenters sent the experts evaluation materials by e-mail, including evaluation description, evaluation criteria, and score sheets. Each expert assessed the twelve life situations based on the following five factors, respectively:

- Occurrence possibility: the possibility of these life situations in which aging adults live alone at home
- Importance: the importance of these life situations to aging adults while they live alone at home
- Possibility of social robots' providing companionship: the possibility of social robots providing companionship for aging adults in this life situation
- Requirements for social robots' companionship: aging adults' requirements for social robots' companionship in this life situation
- Possibility of social robots' reducing loneliness: the possibility that social robots' companionship makes aging adults feel less lonely

The five factors assessed the feasibility of social robots' companionship for aging adults. Among them, two factors assessed the life situations, involving occurrence possibility and importance; three factors assessed social robots' companionship, involving possibility of social robots' providing companionship, requirements for social robots' companionship, and possibility of social robots' reducing loneliness.

The evaluation adopted a five-point Likert scale with “1 = much lower” and “5 = much higher.” In addition, each expert was asked to weigh the five factors and the sum of the weights of the five factors was one. During the evaluation, if experts had any question, they could inquire the experimenters by e-mail.

### 4.2. Results

The score of each life situation by one expert was obtained using a weighted summation method. The total score of each life situation was obtained through the summation of the five experts' scores. The life situations were ranked according to the total scores and were listed in descending order in Table 3—the higher the score, the higher the feasibility of the social robots' companionship for aging adults. The top one-third life situations in the ranking list were considered as high-feasibility ones, involving dining, short breaking, sleeping, and doing housework; this suggested that social robots' companionship had high levels of feasibility for aging adults in these life situations. The middle one-third life situations were considered as medium-feasibility ones, involving doing healthcare, entertaining, watching TV, and reading; this suggested that social robots' companionship had medium levels of feasibility for aging adults in these life situations. The last one-third life situations were considered as low-feasibility ones, involving exercising, studying, using computers/telephones, and doing religious activities; this suggested that social robots' companionship had weak levels of feasibility for aging adults in these life situations.

### 4.3. Comparisons with the Results of Questionnaire Survey

The expert evaluation ranked aging adults' life situations according to the levels of feasibility of social robots' companionship, whereas the questionnaire survey ranked the life situations according to aging adults' demands for companionship. There were differences between the ranking lists of life situations in these two phases as shown in Table 4. The ranking list in questionnaire survey was named experts' ranking list and the one in expert evaluation as aging adults' ranking list. Each ranking list was divided into three levels—high, medium, and low—and each level consisted of four life situations. In one ranking list, the maximum difference of the ranking numbers of two life situations belonging to the same levels was three, and the maximum difference of the ranking numbers of two life situations belonging to two adjacent levels was seven. Hence, from the aspects of social robots' applicability to provide companionship in the life situations, this study considered the life situation of which there was no difference between its ranking numbers in the experts' ranking list and in the aging adults' ranking list to be “highly applicable”, which suggested that there was a greater likelihood that the companionship that aging adults needed in this life situation might be provided by social robots; these life situations involved dinning and watching TV. The life situation of which there were 1, 2, or 3 differences between its ranking numbers in the two ranking lists was considered to be “applicable,” such as doing housework, entertaining, doing healthcare, reading, and doing religious activities; this suggested that there was a likelihood that the companionship that aging adults needed in these life situations might be provided by social robots. In addition, the life situation of which there were 4, 5, 6, or 7 differences between the ranking numbers in the two ranking lists was considered to be “inapplicable,” such as exercising, studying professional skills, and using computer/telephones. The life situation of which there were 8 or above differences between its ranking numbers in the two ranking lists was considered to be “adverse,” such as sleeping and short breaking.

## 5. Discussion

Whether social robots might provide mental consolation to aging adults, such as companionship, remains a problem in robotic science, psychology, and social science. How social robots might replace human beings in some life situations remains a huge challenge for researchers and engineers. This study conducted a questionnaire survey to investigate aging adults' demands for companionship in life situations in which they live alone. In addition, due to the limitations of aging adults' imagination for their interaction with social robots, this study conducted an expert evaluation to investigate the feasibility of social robots' companionship for aging adults in life situations; this made up for the disadvantages of aging adults' imagination and provided objective requirements for companionship. The differences between the results of the questionnaire survey and the expert evaluation reflect both the possibility and the inadequacy that it was social robots to provide companionship to aging adults.

Among the life situations collected in the interview survey, both dinning and watching TV ranked the same in the two ranking lists; dinning ranked No. 1 in both ranking lists and belonged to high-demand/high-feasibility level, whereas watching TV ranked No. 7 in both ranking lists and belonged to medium-demand/medium-feasibility level; this suggests that in both life situations, aging adults need to be accompanied and there was a greater likelihood that the aging adults' demands for companionship might be fulfilled by the companionship provided by social robots. The two life situations are characterized by the fact that aging adults might want to share the feelings of eating or watching with their companions.

In the life situations of doing housework, entertaining, doing healthcare, reading, and doing religious activities, their ranking numbers differed a little in the two ranking list; this suggested that if aging adults needed companionship, social robots were relatively applicable to provide it.

For the lift situations which presented social robots' inapplicability, including exercising, studying professional skills, and using computer/telephones, there are two cases for this inapplicability. One is that the life situation has a lower ranking in the experts' ranking list which was according to the feasibility of social robots' companionship and a higher ranking in the aging adults' ranking list which was according to aging adults' demand for social robots' companionship. In this case, aging adults need companionship but it is not suitable to be provided by social robots; this suggests that human beings' companionship is not replaceable. The other case is that the life situation has a higher ranking in the experts' ranking list but a lower ranking in the aging adults' ranking list. In the second case, it is suitable for social robots to provide companionship but aging adults do not think they need it. There are two explanations for this ranking difference. One explanation is that aging adults do not realize that they need to be accompanied. The other explanation is that the companionship provided by social robots differs from that provided by human beings. Further studies might investigate the differences between companionship provided by social robots and that by human beings.

Both the life situations of sleeping and short breaking ranked adversely in the two ranking lists, and they were both reported by aging interviewees to rank at the low-demand level and assessed by experts to rank at the high-feasibility level. The ranking differences might explain that aging adults are worried about being bothered by social robots while resting, but experts think that aging adults might feel loneliness and need companionship in these situations, and social robots are fully capable of providing companionship. Further studies are required to determine the reasons for the cognition differences.

The applicability of social robots' companionship should be considered during their service design. The companion service should fulfill aging adults' real demands. Multiple methods could provide design suggestions from different aspects.

In addition, based on the results of the interview, most of the aging interviewees, 7 (out of 8), reported that they had ever seen or used robots. All of them had the habits of watching TV or using computers/telephones and they saw robots through TVs, computers, or telephones; this suggests that media is an important channel to spread new technology. Aging adults showed great curiosity and imagination when they were describing robots; this is consistent with the previous research [22].

## 6. Conclusions

The purposes of this study involved (1) collecting aging adults' life situations in which they live alone at home; (2) investigating their demands for companionship in these life situations; (3) investigating the feasibility of social robots' companionship for aging adults in these life situations; and (4) comparing aging adults' demands and the feasibility of social robots' companionship to explore the possibility of replacing human beings' companionship with social robots'. This study involved three phases. Phase 1 interviewed eight aging adults, and twelve life situations in total were collected. Based on the results of Phase 1, a questionnaire survey (Phase 2) involving 234 valid responses and an expert evaluation (Phase 3) involving five experts were conducted to investigate aging adults' demands for companionship and the feasibility of social robots' companionship in these life situations, respectively.

Based on the results of the questionnaire survey, aging adults had a high level of demands for companionship in the life situations of dinning, doing housework, exercising, and doing healthcare. Based on the results of the expert evaluation, social robots' companionship had a high level of feasibility for aging adults in the life situations of dinning, short breaking, sleeping, and doing housework. Based on the comparison between the results of the questionnaire survey and the expert evaluation, aging adults' demands for companionship and the feasibility of social robots' companionship varied a lot—social robots were suitable to provide companionship instead of human beings in the life situations of dinning and watching TV and relatively applicable to provide companionship in the life situation of doing housework, entertaining, doing healthcare, reading, and doing religious activities. In other life situations, social robots' companionship showed inapplicability. This study provided design recommendations for the companion service of social robots and helped to establish a well-developed human-robot interaction system and to provide better home-based elder care services for aging adults.

## Figures and Tables

**Table 1:** Occurrence frequency and demand frequency of life situations mentioned by interviewees.

Life situations	Occurrence frequency	Demand frequency
Dinning	8	7
Doing housework	5	5
Doing healthcare	5	3
Exercising	4	2
Watching TV	4	2
Sleeping	8	1
Using computers/telephones	4	1
Reading	3	1
Religious activities	2	1
Entertaining	3	1
Studying	3	0
Short breaking	2	0

**Table 2:** Ranking of life situations according to aging adults' demands for companionship.

Rank	Life situations	Mean	Standard deviation	Cronbach's alpha	Demand level
1	Dinning	3.77	0.61	0.730	High
2	Doing housework	3.39	0.67	0.823	High
3	Exercising	3.29	0.55	0.743	High
4	Doing healthcare	3.20	0.66	0.809	High
5	Using computers/telephones	3.09	0.69	0.770	Medium
6	Studying	2.92	0.64	0.763	Medium
7	Watching TV	2.91	0.57	0.700	Medium
8	Entertaining	2.88	0.67	0.759	Medium
9	Religious activities	2.87	0.65	0.867	Low
10	Short breaking	2.78	0.82	0.848	Low
11	Reading	2.63	0.73	0.777	Low
12	Sleeping	2.50	0.61	0.716	Low

**Table 3:** Rank of the feasibility of social robots' companionship according to life situations.

Rank	Life situation	Total score of evaluation	Feasibility level
1	Dining	22.15	High
2	Short breaking	20.55	High
3	Sleeping	20.30	High
4	Doing housework	18.87	High
5	Doing healthcare	18.20	Medium
6	Entertaining	17.83	Medium
7	Watching TV	17.27	Medium
8	Reading	14.20	Medium
9	Exercising	13.55	Low
10	Studying	12.57	Low
11	Using computers/telephones	11.90	Low
12	Doing religious activities	10.57	Low

**Table 4:** Ranking differences between life situations in the experts' ranking list and those in the aging adults' ranking list.

Life situations	Experts' ranking (feasibility)	Aging adults' ranking (demand)	Absolute differences between two ranking numbers	Social robots' applicability
Dinning	1	1	0	Highly applicable
Short breaking	2	10	8	Adverse
Sleeping	3	12	9	Adverse
Doing housework	4	2	2	Applicable
Entertaining	5	8	3	Applicable
Doing healthcare	6	4	2	Applicable
Watching TV	7	7	0	Highly applicable
Reading	8	11	3	Applicable
Exercising	9	3	6	Inapplicable
Studying	10	6	4	Inapplicable
Using computers/telephones	11	5	6	Inapplicable
Religious activities	12	9	3	Applicable
